# Feeding Preferences and Responses of *Monochamus saltuarius* to Volatile Components of Host Pine Trees

**DOI:** 10.3390/insects13100888

**Published:** 2022-09-29

**Authors:** Jue Wang, Sufang Zhang, Yanan Zheng

**Affiliations:** 1 College of Forestry, Shenyang Agricultural University, Shenyang 110866, China; 2 Key Laboratory of Forest Protection of National Forestry and Grassland Administration, Ecology and Nature Conservation Institute, Chinese Academy of Forestry, Beijing 100091, China

**Keywords:** *Monochamus saltuarius*, feeding preferences, host volatiles, attractant

## Abstract

**Simple Summary:**

*Bursaphelenchus xylophilus* causes pine wilt disease, which is one of the most devastating forest diseases in the world. However, *B. xylophilus* cannot spread naturally and must be carried by vector insects from infested trees to healthy ones. *Monochamus saltuarius* is a *B. xylophilus* vector that has caused massive pine tree mortality in Japan, South Korea, and China’s middle temperate zone. In China, there have been only a few reports of this species. The feeding preference of *M. saltuarius* on different host pine trees was determined, and the volatile components of the host pine trees were analyzed. Furthermore, the responses of *M. saltuarius* to different host volatiles were determined, and the correlation between *M. saltuarius* preference and host pine tree volatiles was investigated. This study will serve as a reference for the development and application of *M. saltuarius* attractants, which will aid in the control of *M. saltuarius* and pine wilt disease.

**Abstract:**

*Monochamus saltuarius* is a vector of *Bursaphelenchus xylophilus* and has caused massive mortality in pine trees in Japan, South Korea, and the middle temperate zone of China. In this study, the feeding preferences of *M. saltuarius* to four common host pine species in the middle temperate zone of China were investigated in a laboratory. The volatiles of the four host pine species were sampled and analyzed by gas chromatography and mass spectrometry. The responses of *M. saltuarius* to host pine tree volatiles were measured using a four-arm olfactometer. The results showed that: (1) the preference of *M. saltuarius* for *Pine tabulaeformis* was significantly higher than the other ones. (2) The composition and content of the volatiles of the four tested host pine twigs were different, and the relative content of α-pinene was the highest. (3) *M. saltuarius* was highly sensitive to α-pinene, β-pinene, limonene, and 3-carene, and the attracting effect was enhanced by the mixture of volatile components. This study provides a reference for the development and application of attractants for *M. saltuarius*. Our results would be helpful in controlling *M. saltuarius* and pine wilt disease.

## 1. Introduction

*Monochamus saltuarius* (Coleoptera: Cerambycidae) is a species of pinewood borer that primarily damages members of the Pinaceae family. The mechanical damage caused by *M. saltuarius* feeding on pine trees has not been reported to cause serious economic losses. However, *M. saltuarius* is a vector of *Bursaphelenchus xylophilus*, which has caused massive mortality in pine trees [1]. In 1987, for the first time in Japan, it was confirmed that *M. saltuarius* is a vector insect of *B. xylophilus* [2,3]. that primarily infested *Pinus densiflora* and *P. thunbergii* [4]. In South Korea, *M. saltuarius* has also been found to transmit *B. xylophilus* [5], which mainly infests *P. koraiensis* [5,6]. In 2018, *M. saltuarius* was confirmed as a vector insect of *B. xylophilus* in the middle temperate zone of China [7].

The damage caused by the longhorn beetle to the host tree is hidden and difficult to control. Among the existing control technologies, trapping is recognized as one of the most effective. Trapping can also monitor the occurrence of a population, which is helpful for the prediction of pest populations [8]. At present, attractants have been widely used in the control of *M. alternatus*, which is also a vector of *B. xylophilus*. In particular, the compound attractant composed of pheromone and host pine tree volatiles has a significant trapping effect on *M. alternatus*, and has become the main measure to monitor and control *M. alternatus* [9]. Host volatiles are important substances that play a role in attracting adults during the supplementary nutrition period [10]. The composition and relative content of volatiles in different host plants are different. The main hosts of *M. alternatus* are *P. massoniana*, *P. thunbergii*, and *P. taeda* [1]. In contrast, the main hosts of *M. saltuarius* are *P. koraiensis*, *P. tabulaeformis*, *P. sylvestris* var. *mongholica,* and *Larix* spp. [11,12,13]. At present, there are only a few studies on the attractant of *M. saltuarius*. These studies reported that the main component of the aggregated pheromone of *M. saltuarius* was 2-undecyloxy-1-ethanol [14]. The forest experiments showed that mixed attractants of 2-undecyloxy-1-ethanol, α-pinene and ethanol had a significant trapping effect on *M. saltuarius* [15,16]. The feeding preference of *M. saltuarius* for host pine trees and the volatile components of host pine trees need to be further studied.

In this study, the feeding preference of *M. saltuarius* on different host pine trees was determined, the volatile components of the host pine trees were analyzed, and differences in their composition were compared. Additionally, the responses of *M. saltuarius* to different host volatiles were determined, and the correlation between the preference of *M. saltuarius* and host pine tree volatiles was studied. This study will provide a reference for the development and application of attractants for *M. saltuarius*, which will be helpful in controlling *M. saltuarius* and pine wilt disease.

## 2. Materials and Methods

### 2.1. Insects and Plants

*M. saltuarius* larvae were collected in October 2021 from Dahuofang Forest in Fushun (41°56′16.3″ N, 124°13′6.5″ E, 173.1 m), Liaoning, China. *P. koraiensis* infected by *M. saltuarius* was cut down, and then *M. saltuarius* larvae were collected by splitting the xylem and taken to the laboratory. *M. saltuarius* larvae were reared in an incubator under the following conditions: temperature of 24 ± 2 °C, relative humidity (RH) of 55 ± 5%, and photoperiod of 16L: 8D. *M. saltuarius* emerging adults were checked and collected every day. One-year-old twigs of *P. koraiensis*, *P. tabulaeformis*, *P. sylvestris* var. *mongholica*, and *Larix olgensis* were collected from the Dahuofang Forest. 

### 2.2. Chemicals

Seven chemicals were chosen based on the results of GCMS analysis of the volatile components of the host pine tree and previous studies [15,16]. All chemicals used in this work were purchased from J&K Scientific (Beijing, China), including α-pinene, ≥99%, CAS 3856–25–5; β-pinene, ≥98%, CAS 127–91–3; limonene, ≥99%, CAS 5138–86–3; α-caryophyllene, ≥95%, CAS 6753–98–6; β-phellandrene, ≥98%, CAS 99–83–2; bornyl acetate, ≥99%, CAS 5655–61–8; 3-Carene, ≥99%, CAS 13466–78–9; as well as ethanol (EtOH), 200 proof HPLC grade and paraffin liquid, HPLC grade. They were used without further purification. Paraffin liquid was used as a solvent for dilution when lower stock concentrations of any of the aforementioned chemicals were required.

### 2.3. Determination of Feeding Preference for Different Host Pine Trees

Four kinds of pine twigs were placed in the four corners of the insect’s cage (60 cm × 120 cm × 120 cm). In addition, newly emerging *M. saltuarius* adults (five male adults and five females) were placed in the insect cage (temperature 24 ± 2 °C, relative humidity 55 ± 5%, photoperiod 16L: 8D), and six replicates were tested for a four-choice test. To ensure that adults had sufficient food sources, pine twigs were replaced every third day. To facilitate statistics, the feeding amount of adults was calculated according to the feeding area. When shoots and buds were fed, the length and width of the feeding amount were the length and width of the shoots and buds, respectively (mm). When fed on the needles, the width of the feeding amount was recorded as one millimeter. The feeding amount was counted once a day for a total of 10 days.

Feeding amount (mm^2^) = length of feeding part (mm) × width of feeding part (mm);

Total feeding amount (mm^2^) = total feeding amount of needles (mm^2^) + total feeding amount of shoots (mm^2^) + total feeding amount of buds (mm^2^);

Feeding ratio (%) = total feeding amount to feed on one host pine tree (mm^2^)/total feeding amount (mm^2^) × 100%.

### 2.4. Host Pine Tree Volatile Collections and GC-MS Spectrometry

The twigs of *P. koraiensis*, *P. tabulaeformis*, *P. sylvestris* var. *mongholica*, and *L. olgensis* were cut in the forest and packed in a microwave bag (Reynolds, New York, NY, USA, 44.3 cm × 55.8 cm). Air in the bag was extracted, and bottled air was injected. Then, the two ends were connected to a hollow glass tube with activated carbon and an adsorbent. The sampling time was 40 min, with a flow rate of 100 mL/min. The extracted adsorption tube was covered with a polytetrafluoroethylene bottle cap and stored at −80 °C. The adsorbed volatiles were eluted in a two-milliliter sample bottle (Agilent, Santa Clara, CA, USA) with 600 µL of chromatographic grade redistilled n-pentane in the laboratory for Gas Chromatography Mass Spectrometer (GC-MS) analysis. The eluted volatile substances were injected into two microliters on GC-MS (Agilent 19091S-433E). A spectral library (NIST11) and standards were used for characterization, and an internal standard method was used for quantification (the internal standard compound was ethyl heptanoate). The chromatographic column was DB-WAX (60 m × 0.25 mm × 0.25 µm), and the temperature was programmed at 30 °C for 2 min, and then increased to 200 °C at a rate of 5 °C/min. The inlet temperature was set to three gradients of 180 °C, 200 °C, and 220 °C. The carrier gas was high-purity He (99.999%), and the carrier gas flow rate was 1 mL/min. The ionization method was EI; the ionization energy was 70 eV; the temperature of the ion source was 230 °C; the temperature of the quadrupole was 150 °C; the scanning mass range was 30–300 amu.

### 2.5. Four-Arm Olfactometer Test

The basic composition and connection order of the four-arm olfactometer is suction pump → flowmeter → activated carbon drying tower → flavor source bottle → four-arm olfactometer. In order to determine the response of *M. saltuarius* to different compounds of host pine tree volatiles, each compound was diluted 10, 100, and 1000 times in paraffin liquid, and 20 µL of the diluted compounds was applied to a filter paper. After the 30 s, the samples were placed in bottles, and filter paper coated with an equal amount of paraffin liquid was placed in another bottle. To determine the response of *M. saltuarius* to different components of host pine tree volatiles, 4 compounds (α-pinene, β-pinene, limonene, 3-carene) were mixed into Component I, 8 compounds (α-pinene, β-pinene, limonene, 3-carene, α-caryophyllene, β-phellandrene, bornyl acetate, anhydrous ethanol) were mixed into Component Ⅱ. The method of measuring the response of *M. saltuarius* to the two components was the same as that of a single compound.

Healthy *M. saltuarius* adults were selected after 6 h of starvation treatment. They were placed into the test chamber after the four-arm olfactometer flowmeter was stable at 1.5 L/min. At room temperature (25 ± 1 °C) condition, the four-arm olfactometer began pumping at the selected timing for 10 min. The number of *M. saltuarius* staying in the trap bottles and in different areas was recorded. After each determination, the test chamber was scrubbed with 75% ethanol and rotated 90°, and 50 replicates were tested for each treatment (25 male adults and 25 female adults). Compounds highly sensitive to *M. saltuarius* were selected and prepared according to natural proportions to determine the sensitivity of *M. saltuarius* to different volatile components.

The reaction rate (%) = total number of *M. saltuarius* in trap bottle/total number of tested *M. saltuarius* × 100%

Selection coefficient = (number of *M. saltuarius* in treatment bottle − number of *M. saltuarius* in control bottle)/(number of *M. saltuarius* in treatment bottle + number of *M. saltuarius* in control bottle).

### 2.6. Statistics

Data analysis was performed with IBM SPSS statistics 22.0 software (IBM Analytics, New York, NY, USA). Data from bioassays that evaluated the *M. saltuarius* response to treatment trap tubes versus control trap tubes were converted to percentages and analyzed using Tukey tests and the Chi-square test.

## 3. Results

### 3.1. Feeding Preference on Different Host Pine Trees

**Figure 1:**
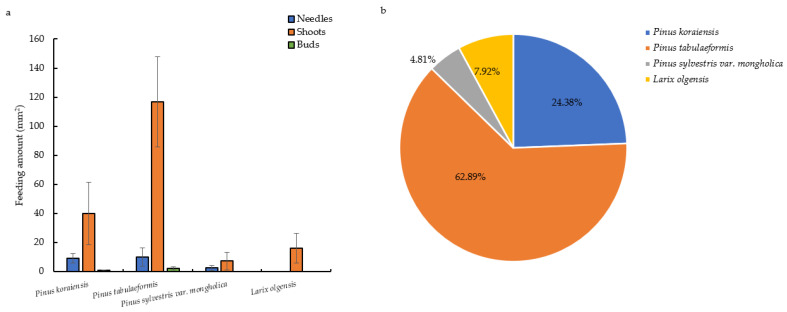
Feeding preference of *Monochamus saltuarius* adults on different host pine trees. (**a**) Average daily feeding amount of *Monochamus saltuarius* adults on different host pine trees; (**b**) Feeding ratio of *Monochamus saltuarius* adults on different host pine trees.

The daily feeding amount of *M. saltuarius* adults on *P. tabulaeformis* was the highest (129.14 ± 50.23 mm^2^), which is significantly higher than the other three host pines (F = 17.289, df = 3.20, *p* > 0.05). The daily feeding amount of twigs of *P. tabulaeformis* was also higher than that of the other three host pine species (F = 12.986, df = 3.20, *p* < 0.05) (Figure 1a, Supplementary Data S1). The feeding ratios of *M. saltuarius* adults on four host pine species were *P. tabulaeformis* (62.89%) > *P. koraiensis* (24.38%) > *L. olgensis* (7.92%) > *P. sylvestris* var. *mongholica* (4.81%). The data were analyzed using the Chi-square test, and the results showed that the feeding preference of *M. saltuarius* on the four host pine trees was significantly different, with x^2^ = 85.360, *p* < 0.05 (Figure 1b, Supplementary Data S1).

### 3.2. Analysis of Volatile Compounds in Different Host Pine Trees

**Figure 2:**
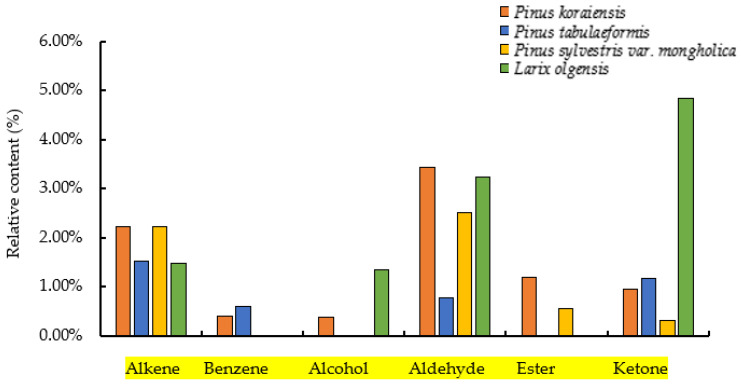
Volatile composition of different host pine trees.

Among the volatile compounds, the relative content of α-pinene was the highest, at 9.26%, 21.29%, 46.16%, and 10.53% in the twigs of *P. koraiensis*, *P. tabulaeformis*, *P. sylvestris* var. *mongholica*, and *L. olgensis*, respectively (Table 1). Twenty compounds were determined from the twigs of *P. koraiensis*, including eight kinds of alkenes, two kinds of benzenes, two kinds of aldehydes, three kinds of alcohols, four kinds of ketones, and one kind of ester. Fifteen different kinds of compounds were determined in *P. tabulaeformis*, including eight kinds of alkenes, three kinds of benzenes, one kind of aldehydes, two kinds of ketones, and one kind of phenols. Seventeen compounds were determined from the twigs of *P. sylvestris* var. *mongholica*, including nine kinds of alkenes, two kinds of aldehydes, one kind of ketone, and five kinds of esters. Twelve compounds were determined from the twigs of *L. olgensis*, including six alkenes, two alcohols, two aldehydes, and two ketones (Figure 2, Supplementary Data S1).

### 3.3. Responses to Host Pine Tree Volatiles

#### 3.3.1. Responses to Different Species of Host Pine Tree Volatiles

**Figure 3:**
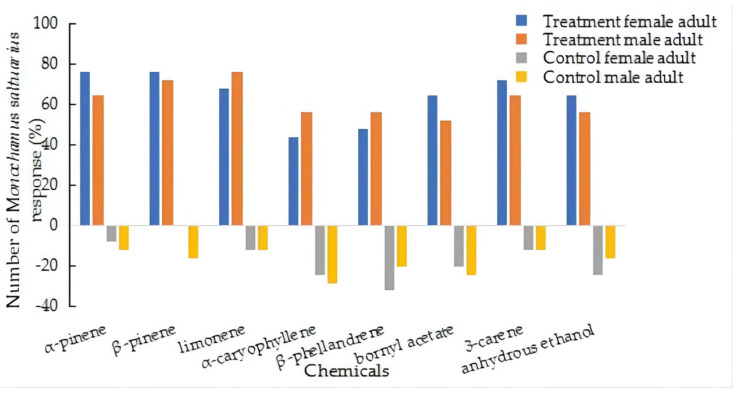
Response of *Monochamus saltuarius* to different host pine tree volatiles.

Among the volatiles of different kinds of host pine trees, *M. saltuarius* has a strong response to α-pinene, β-pinene, limonene, and 3-carene. The selection coefficients were higher than 50% (which were 80.49%, 75.00%, 70.73% and 70.00%, respectively). The responses for α-caryophyllene, β-phellandrene, bornyl acetate, and absolute ethanol were weak, and the selection coefficients were lower than 50% (31.58%, 33.33%, 45.00%, and 48.78%, respectively) (Figure 3, Supplementary Data S1).

#### 3.3.2. Responses to Different Concentrations of Host Pine Tree Volatiles

**Figure 4:**
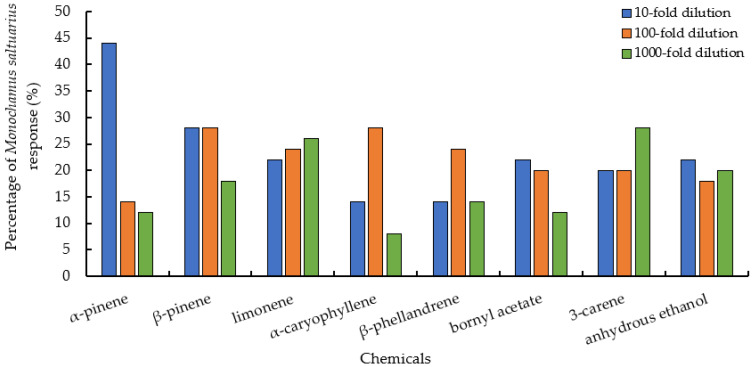
Response of *Monochamus saltuarius* to different concentrations of host pine tree volatiles.

The concentration of host pine volatiles also affected the responses of *M. saltuarius*, with different volatile concentrations when *M. saltuarius*’s response was the strongest. The response of α-pinene, β-pinene, bornyl acetate, and absolute ethanol was higher at 10-fold dilution, to α-caryophyllene and β-phellandrene at 100-fold dilution, and to limonene and 3-carene at 1000-fold dilution (Figure 4, Supplementary Data S1).

#### 3.3.3. Responses to Different Components of Host Pine Tree Volatiles

The results showed that with increasing dilution, the responses of *M. saltuarius* became weaker. It happens in a case when four compounds with strong chemotaxis (α-pinene, β-pinene, limonene, 3-carene) and eight test compounds (α-pinene, β-pinene, limonene, 3-carene, α-caryophyllene, β-phellandrene, bornyl acetate, anhydrous ethanol) were mixed according to the natural proportions. Compared with component Ⅰ, *M. saltuarius* had a stronger response to component Ⅱ, indicating that although *M. saltuarius* was more sensitive to the other four compounds (α-caryophyllene, β-phellandrene, bornyl acetate, anhydrous ethanol); the single component had a weaker response. However, mixing had a synergistic effect on attracting *M. saltuarius* (Table 2).

## 4. Discussion

The research showed that (1) the feeding preference of *M. saltuarius* on *P. tabulaeformis* was significantly stronger than that of *P. koraiensis*, *P. sylvestris* var. *mongholica*, and *L. olgensis*. (2) Concerning the composition and content of the volatiles, there are differences in the proportions, with α-pinene content being the highest. (3) *M. saltuarius* has a strong tropism to single components of α-pinene, β-pinene, limonene, and 3-carene, which were enhanced after mixing. The other compounds with a weaker single response had a synergistic effect after mixing.

Plant volatile compounds include organic compounds, such as hydrocarbons and alcohols. Mixing different volatile compounds in a certain proportion can stimulate the olfactory sense of herbivorous insects and attract insects to feed [17]. The “three-step positioning hypothesis” of the Longhorn beetle [18] indicates that host plant volatiles play an important role in a series of longhorn beetle behaviors, such as feeding, mating, and oviposition. The volatiles of pine needles (α-pinene, β-pinene, and myrcene) are proven attracters of longhorn beetles. Among these monoterpenes, α-pinene has the strongest attracting effect [19]. It has also been observed that the relative content of α-pinene decreases with a decline in pine health [9,20]. 

In this study, the relative content of α-pinene in the four host pine trees was highest, and *M. saltuarius* showed a strong response to α-pinene. Therefore, the preference of *M. saltuarius* for healthy pine twigs may be related to the high content of α-pinene in the twigs. The composition and content of host plant volatiles are closely related to growth status (plant age, developmental status, genetic characteristics, etc.). They are affected by environmental conditions (light, temperature, moisture, nutrition, CO2 concentration, air humidity, etc.), while insect feeding and mechanical damage could also have an impact on host plant volatiles [21]. Therefore, the volatile components of the host pine and their tropism of *M. saltuarius* under different conditions need to be further investigated.

Compared with *M. saltuarius*, the study on *M. alternatus’* response to host volatiles is more comprehensive. Studies have shown that *M. alternatus* mainly relies on sensing various volatiles released by host plants, especially terpenes, to locate the host pine [22]. The volatiles of the host plant can stimulate the feeding habits of *M. alternatus* in either way. Some compounds stimulate its feeding, while some stimulate the antifeeding phenomenon [23]. Adults of *M. alternatus* have different sensitivities and selectiveness to hosts in different physiological states [24]. Simultaneously, mature females prefer to lay eggs on weaker host pine trees. The smaller the relative ratio of α-pinene and β-pinene, the weaker the host tree [25]. Thus, it appears that α-pinene, β-pinene, and β-phellandrene are likely to be the most important signal substances affecting and regulating the feeding and spawning behaviors of *M. alternatus* [26]. In addition, 3-carene, limonene, and longifene also stimulate *M. alternatus* to produce significant antenna potential responses [27,28]. 

In this study, *M. saltuarius* showed stronger tropism to the volatiles (e.g., α-pinene, β-pinene, limonene, and 3-carene) from the host pine, which was similar to the tropism of *M. alternatus*. In addition, there are many types of host plant volatiles, but those few semiochemicals that have a directional effect on insects are often composed of alcohols, aldehydes, ketones, acids, esters, and terpenes. The components are realized in specific concentration ratios [29], and each insect has an optimal dose range for the response of volatile semiochemicals [23]. Although the amount of volatile semiochemicals released by the host plants varies greatly, the levels of each component further vary widely. However, the relative proportions are quite stable [30]. This study showed that the host volatiles of *M. saltuarius* were lower than the host volatiles of the mixed components. Further, we noted that compounds with a weaker tropism of single components have a synergistic effect, which confirms that host volatiles are not caused by themselves. A single component plays a decisive role, but a combination of multiple compounds interacts synergistically. However, compared with component I, there was no significant difference in the attracting effect of component II on *M. saltuarius*, and the cost increased. To take into account the effect and cost of control, component I can be used to develop the attractant of *M. saltuarius* in the future, which has good application prospects.

This study clarified the feeding preference of *M. saltuarius* to common host pine in the middle temperate zone of China, analyzed the composition of host pine tree volatiles, and determined the responses of *M. saltuarius* to host volatiles. This study will provide a reference for the development of *M. saltuarius* attractant and will be helpful in the monitoring and control of *M. saltuarius*. 

## Figures and Tables

**Table 1:** Composition and relative contents of volatile compounds of different host pine trees.

No.	*Pine koraiensis*		*Pine tabulaeformis*		*Pine sylvestris* var. *mongholica*		*Larix olgensis*	
	Chemicals	Relative Content (%)	Chemicals	Relative Content (%)	Chemicals	Relative Content (%)	Chemicals	Relative Content (%)
1	α-pinene	9.26	α-pinene	21.29	α-pinene	46.16	α-pinene	10.53
2	3-ethylbenzaldehyde	5.74	β-pinene	2.58	d-limonene	5.35	5-hydroxy-4-octanone	5.28
3	β-pinene	4.06	1-tetradecene	2.06	2-tetradecene	3.86	3-ethylbenzaldehyde	4.76
4	3-carene	3.66	2-tetradecene	1.86	3-ethylbenzaldehyde	3.64	4-ethylacetophenone	4.41
5	4-ethylacetophenone	3.21	4-Hydroxy-3-methylacetophenone	1.73	camphene	3.13	2-tetradecene	2.44
6	myrcene	2.51	sabinene	1.47	β-pinene	2.73	β-pinene	2.22
7	2-ethylbenzaldehyde	2.31	1,4-diacetylbenzene	1.38	sabinene	1.68	4-ethylbenzaldehyde	1.70
8	4-isopropylbenzyl alcohol	2.27	3-nitrostyrene	1.23	4-ethylacetophenone	1.66	sabinene	1.69
9	limonene	1.95	2-tert-butyl-4-methylphenol	1.19	4-ethylbenzaldehyde	1.41	cis-3-hexen-1-ol	1.44
10	camphene	1.70	camphene	0.78	myrcene	1.31	1,3-dimethylcyclopentanol	1.28
11	1-dodecene	1.37	1,4-diethylbenzene	0.63	3-carene	1.06	camphene	0.52
12	di(2-ethylhexyl)phthalate	1.36	1,5,6,7-tetrahydro-4 h-indol-4-one	0.62	4-carene	0.69	3-carene	0.51
13	1,2-diethylbenzene	0.49	5-nitro-m-xylene	0.55	butyl acetate	0.58		
14	2-hexanol	0.40	4-nitrostyrene	0.54	L-bornyl acetate	0.58		
15	1,4-diethylbenzene	0.29	3-ethylbenzaldehyde	0.41	butyl acrylate	0.63		
16	1-ethenyl-3-ethylbenzene	0.27			butyl propionate	0.44		
17	3-hexanol	0.24						
18	4-ethylpropiophenone	0.22						
19	3-hexanone	0.21						
20	2-hexanone	0.18						

**Table 2:** Reactions of *Monochamus saltuarius* to different mixed components of host pine tree volatiles.

Chemical Compound	Dilution Ratio			CK	No-Response	Reaction Rate	Selection Coefficient
	10	100	1000				
Component I	9 Female 5 Male	6 Female 7 Male	4 Female 3 Male	2 Female 2 Male	4 Female 8 Male	76%	78.95%
Component Ⅱ	11 Female 9 Male	5 Female 4 Male	3 Female 3 Male	1 Female 2 Male	5 Female 7 Male	76%	84.21%

Component I (4 compounds): α-pinene, β-pinene, limonene, 3-carene; Component Ⅱ (8 compounds): α-pinene, β-pinene, limonene, 3-carene, α-caryophyllene, β-phellandrene, bornyl acetate, anhydrous ethanol.

## Data Availability

Data are contained within the Supplementary Material. The data presented in this study are available in Supplementary Materials.

## Supplementary Materials

The following supporting information can be downloaded at: https://www.mdpi.com/article/10.3390/insects13100888/s1.
